# Exploring the Regulation of Jiangtang Tiaozhi Formula on the Biological Network of Obese T2DM Complicated With Dyslipidemia Based on Clinical Transcriptomics

**DOI:** 10.3389/fendo.2022.817147

**Published:** 2022-07-25

**Authors:** Tingting Bao, Song Wang, Yingying Yang, Lisha He, Lin Han, Tiangang Zhai, Jia Chen, Qiang Zhou, Xiyan Zhao, Fengmei Lian, Linhua Zhao, Xiaolin Tong

**Affiliations:** ^1^ Graduate College, Beijing University of Chinese Medicine, Beijing, China; ^2^ Institute of Metabolic Diseases, Guang’ anmen Hospital, China Academy of Chinese Medical Sciences, Beijing, China; ^3^ Department of Geriatrics, First Teaching Hospital of Tianjin University of Traditional Chinese Medicine, Tianjin, China; ^4^ National Clinical Research Center for Chinese Medicine Acupuncture and Moxibustion, First Teaching Hospital of Tianjin University of Traditional Chinese Medicine, Tianjin, China; ^5^ Medical History Teaching and Research Department, Chengdu University of Traditional Chinese Medicine, Chengdu, China; ^6^ Graduate College, Gansu University of Chinese Medicine, Lanzhou, China; ^7^ Beijing Hospital of Traditional Chinese Medicine, Capital Medical University, Beijing, China

**Keywords:** type 2 diabetes mellitus, long non-coding RNA, Jiangtang Tiaozhi recipe, obesity, dyslipidemia, metabolic syndrome

## Abstract

**Objective:**

To use systems biology to explore the biomolecular network mechanism of the Jiangtang Tiaozhi Recipe (JTTZR) in the intervention of obese Type 2 diabetes (T2DM) patients with dyslipidemia.

**Methods:**

Twelve patients with obese type 2 diabetes mellitus and dyslipidemia (traditional Chinese medicine syndrome differentiation was excess heat syndrome of the stomach and intestines) were treated with JTTZR for 24 weeks, and 12 patients were included in the healthy control group. First, blood samples from 6 patients in each group (disease group before treatment, disease group after treatment, and healthy control group) were collected for RNA microarray analysis. Quantitative polymerase chain reaction (qPCR) was used to validate these target lncRNAs and mRNAs. Finally, a detailed analysis of the differences in the disease group before treatment vs. the healthy control group and the disease group after treatment vs. the disease group before treatment was undertaken. In addition, we focused on disease-related pathways and analyzed the correlation between the differential expression of target lncRNAs and clinical indicators.

**Results:**

(1) Disease group before treatment vs. healthy control group: There were 557 up-regulated lncRNAs, 273 down-regulated lncRNAs, 491 up-regulated mRNAs, and 1639 down-regulated mRNAs. GO analysis and pathway analysis showed that T2DM may be related to cell proliferation in the forebrain, post-embryonic organ development, calcium signaling pathway. qPCR validation showed that the expression of XLOC-005590 and HNF1A-AS1 as target lncRNAs increased, and this was verified by gene chip analysis. (2) Disease group after treatment vs. disease group before treatment: 128 lncRNAs were upregulated, 32 lncRNAs were downregulated, 45 mRNAs were upregulated, and 140 mRNAs were downregulated. GO analysis and pathway analysis showed that JTTZR may treat T2DM through endosome transport, the insulin signaling pathway, and glycine, serine, and threonine metabolism. qPCR validation showed that in the healthy control group, XLOC_005590 was upregulated, whereas the downstream gene (ECI2) was downregulated in the disease group before treatment. However, after 24 weeks of intervention with JTTZR, XLOC_005590 was downregulated and ECI2 was upregulated compared with the disease group before treatment (0 weeks) (*P <*0.05).

**Conclusion:**

JTTZR may interfere in patients with obese T2DM with dyslipidemia by regulating pathways such as fatty acid degradation, glycolysis/gluconeogenesis, and pyruvate metabolism.

## Introduction

**Figure 1:**
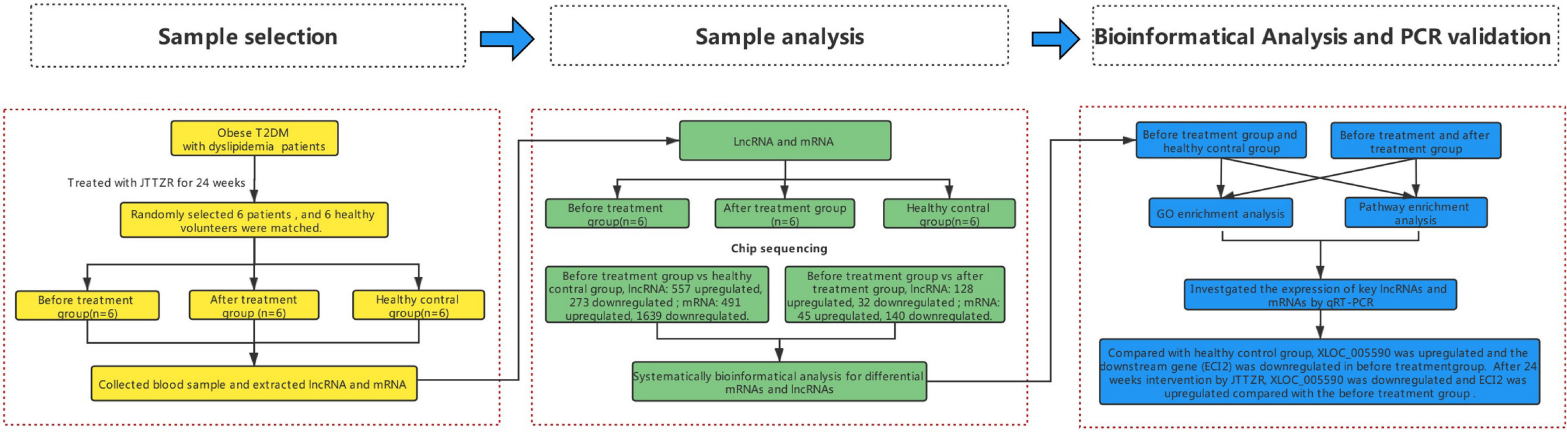
The research processes.

Obese type 2 diabetes mellitus (T2DM) with dyslipidemia is very common in clinics. Modern medicine uses treatment measures, such as glucose reduction, lipid reduction, and weight loss, for each pathological component. The multi-channel, multi-target, and more individualized treatments of Chinese medicine formulas have significant clinical effects. Based on the traditional Chinese medicine (TCM) theory and years of clinical experience, Professor Tong Xiaolin proposed the theory of *ointment turbidity*, aiming at the homology of sugar, lipid, and fat in metabolic syndrome. In this study, under the guidance of the theory of *ointment turbidity*, the Dahuang Huanglian Xiexin Decoction was used to form the Jiangtang Tiaozhi Recipe (JTTZR), which was used to treat patients with newly diagnosed type 2 diabetes with dyslipidemia. Previous studies have shown that JTTZR can reduce glycosylated hemoglobin, blood sugar, blood lipids, body weight, waist circumference, and hip circumference, and improve islet cell function and insulin resistance (1). It is preliminarily proven that the Jiangtang Tiaozhi prescription can simultaneously regulate sugar, lipid, and obesity. In this study, 12 obese T2DM patients with dyslipidemia (*stomach-intestines excess heat syndrome*) were selected for JTTZR intervention, and 12 patients were included in a healthy control group. The lncRNA gene chip and subsequent quantitative polymerase chain reaction (qPCR) verification were used to elucidate the mechanism of JTTZR and identify potential biomarkers. The research process is shown in **Figure 1**.

## Materials and Methods

### Research Design

#### Research Object

This clinical trial was registered at Clinicaltrials.gov (NCT04623567). Patients with first-onset obese T2DM with dyslipidemia (excess heat syndrome of the stomach and intestines) were admitted to Guang’anmen Hospital of China Academy of Chinese Medical Sciences (2012.2 -2012.7). JTTZR is composed of *Anemarrhenae rhizoma*, *Momordica charantia*, *Coptis chinensis*, *Salvia miltiorrhiza*, *red yeast rice*, *Aloe vera*, *Schisandra chinensis*, *and dried ginger* (**Table 1**). In our previous study, we confirmed eight major compounds: mangiferin, coptisine, jatrorrhizine, salvianolic acid B, aloin, berberine, palmatine, and lovastatin (2). Each patient received 30 g JTTZR granules daily. The JTTZR intervention consisted of one 30 g dose twice a day over 24 weeks. All drugs used were produced as a single batch and passed inspection. The glucose and lipid metabolism indices, such as glycosylated hemoglobin, fasting blood glucose, two hours postprandial blood glucose, and blood lipids, were tested before and after treatment. The obesity-related indicators, such as weight, waist circumference, and body mass index, were also recorded. From these patients, 12 were selected for the study as they met the following criteria: 1) HbA1c (0 weeks) ≥ 7% and HbA1c (24 weeks) ≤ 6.5%, or 2)6.5% ≤ HbA1c (24 weeks) ≤ 7%, but HbA1c (0 weeks)-HbA1c (24 weeks) ≥1. Twelve healthy people were included as the control group, and there was no statistical difference in sex and age (30–75 years old). The blood samples of patients in the healthy control group, disease group at 0 weeks, and disease group at 24 weeks were stored at –80°C for subsequent detection and analysis of lncRNA expression differences. This study was approved by the Ethics Committee of Guang’anmen Hospital of China Academy of Chinese Medical Sciences. All the participants provided written informed consent.

**Table 1:** The compostion of JTTZR.

Chinese Name	Herb Name	Latin Name	Weight*	Family
Zhi-mu	Anemarrhenae rhizoma	*Anemarrhena asphodeloides* Bunge	7.5	Asparagaceae
Ku-gua	Momordica charantia	*Momordica charantia* L.	6	Cucurbitaceae
Huang-lian	Coptidis rhizoma	*Coptis chinensis* Franch.	2.5	Ranunculaceae
Dan-shen	Salviae miltiorrhizae radix et rhizoma	*Salvia miltiorrhiza* Bunge	1.8	Lamiaceae
Hong-qu	Red yeast rice		6	
Lu-hui	Aloe	*Aloe vera *(L.) Burm.f.* *	3	Asphodelaceae
Wu-wei-zi	Schisandrae chinensis fructus	*Schisandra chinensis* (Turcz.) Baill.	1	Schisandraceae
Gan-jiang	Zingiberis rhizoma	*Zingiber officinale* Roscoe	1	Zingiberaceae

Red yeast rice is a Chinese medicine fermented by rice and Monascus purpureus Went. Therefore, there are no corresponding Latin names or families. *Ratio between Herbs.

#### Inclusion Criteria

1. The patient signed the informed consent.
2. Waist circumference ≥90 cm for men and ≥80 cm for women.
3. According to the 1999 WHO standard, newly diagnosed and treated patients met the diagnostic criteria for T2DM. After the screening period (diet control + exercise therapy for 4 weeks), fasting blood glucose ≥7.0 mmol/L, but fasting blood glucose <13.9 mmol/L, or 2 h postprandial blood glucose ≥11.1 mmol/L; and glycosylated hemoglobin ≥7.0%.
4. After the screening period (diet control + exercise therapy for four weeks), triglyceride (TG) ≥ 1.7 mmol/L.
5. TCM syndrome differentiation was excess heat syndrome of the stomach and intestines (refers to the “Guidelines for the Prevention and Treatment of Diabetes by Traditional Chinese Medicine”): (1) The main symptoms are epigastric distention, discomfort due to bloating, and constipation. (2) Secondary symptoms: dry mouth, bitter taste, or bad breath; thirst; a liking for cold drinks; overeating and hunger; red tongue and yellow coating; and fast pulse. (3) The patient had one main symptom and two secondary symptoms.
6. Age: 30–70 years old.

#### Exclusion Criteria

1. Use of insulin therapy. Those who had been treated for diabetes (including other Chinese and Western medicines, physical therapy, psychological therapy, and health food) for more than 3 months in the past. Those who were treated with hypoglycemic and lipid-lowering drugs within one month before enrollment.
2. Those with diabetic complications as the main symptoms, and those with severe heart, lung, liver, kidney, brain, and other complications, or combined with other serious primary diseases.
3. Uncontrolled or controlled blood pressure, systolic blood pressure ≥ 160 mmHg or (and) diastolic blood pressure ≥ 100 mmHg.
4. Diabetic ketosis, ketoacidosis, and severe infections within the past month.
5. Mental illness.
6. Pregnant women are those planning on becoming pregnant or breastfeeding.
7. Those who are allergic to the ingredients of this traditional Chinese medicine; and those who have allergies.
8. Patients who participated in other drug clinical studies or participated in other clinical studies within 1 month before participating in this study.
9. Those with alcohol, drug, and/or psychoactive substance dependencies within the past five years.
10. Patients with other lesions or conditions that reduce the likelihood of enrollment or complicate enrollment according to the investigator’s judgment. These include frequent changes in the working environment, an unstable living environment, and other situations that are likely to result in loss of follow-up.
11. Instability in the dosage and type of antihypertensive drugs used.
12. Those who consume medicines or health foods that affect body weight.
13. Patients with impaired liver and kidney function (ALT and AST levels greater than double the upper limit of normal values). The serum creatinine level was greater than the upper limit of normal values.
14. Insensitivity to hypoglycemia.

### Experimental Materials

#### Main Reagents and Instruments

TRI reagent BD (MRCGENE), isopropanol, and 100% ethanol were obtained from Shanghai Chemical Reagent Co., Ltd., and glacial acetic acid from Sinopharm Group Chemical Reagent Co., Ltd. RNase-free glycogen, SuperScript” III Reverse Transcriptase, and 5× RT buffer were obtained from Invitrogen Life Technologies, and RNase inhibitor from Epicenter Inc. dNTP mixture (dATP, dGTP, dCTP, and dTTP, 2.5 mM) were obtained from HyTest Ltd. Primer (HyTest Ltd.).

A Clean Bench (Shanghai Bo Xun Industrial Co. Ltd. Medical Equipment Factory), DK-8D electric heating constant-temperature water tank (Shanghai Senxin Experimental Instrument Co., Ltd.), and a Gene Amp PCR System 9700 (Applied Biosystems) were used forLncRNA gene chip detection was performed using an Arraystar human lncRNA chip V3.0 chip.

### Experimental Methods

#### LncRNA Gene Chip Detection

Blood sample (200 µL), TRIREAGENT BD (750 μL) and 20 μL xxx were added to a centrifuge tube containing 1.5 mL glacial acetic acid and mixed. The homogenized sample was incubated at 15–30°C for 5 min to completely dissociate the nucleic acid-protein complex. Each 750 μL of TRI Reagent BD–homogenized sample was mixed with 0.2 mL of chloroform and incubated at 15–30°C for 2–3 min. The samples were then centrifuged at 12,000 × *g* for 15 min at 4°C. After centrifugation, the mix was divided into a lower red phenol-chloroform phase, middle layer, and upper colorless water phase. RNA was distributed throughout the water phase. The volume of the water phase was approximately 60% of the volume of TRI Reagent BD added during homogenization. The aqueous phase was transferred to a new centrifuge tube, 500 μl isopropanol was added, and mixed to precipitate RNA. After mixing, the mixture was incubated at 15–30°C for 10 min and centrifuged at 12,000 × *g* at 4°C for 10 min. At this time, the RNA precipitate that was not visible before centrifugation formed a gelatinous precipitate on the bottom and sidewalls of the tube. After washing, the RNA pellet was re-dissolved. A NanoDrop ND-1000 was used to check the quality of the extracted RNA, and RNA integrity was assessed by standard denaturing gel electrophoresis. RNA labeling and gene chip hybridization were performed by Shanghai Kangcheng Biotechnology Co. Ltd. The Arraystar human lncRNA chip V3.0 chip encoding transcripts can detect 30586 lncRNAs and 26109 proteins. The lncRNAs involved in the test were selected from authoritative public transcriptome databases (including Refseq, UCSC knowledge genes, and Gencode) and high-impact factor papers. Sample labeling and chip hybridization were performed according to the Agilent One-Color Microarray-based Gene Expression Analysis protocol (Agilent Technology).

The Agilent Feature Extraction software (v11.0.1.1) was used to obtain the chip map and read the value to obtain the original data. GeneSpring GX v12.1 software (Agilent Technologies) was used to standardize the raw data and subsequent data processing. After standardization of the raw data, high-quality probes were screened (at least five of the 30 samples of a probe were labeled as present or marginal) for further analysis. Statistically significant differentially expressed lncRNAs or differentially expressed mRNAs between the two groups of samples were screened by *P*-value/FDR. Differentially expressed lncRNAs or mRNAs between the two samples were screened using fold change (FC).

#### qPCR Verification of Target lncRNAs and Related mRNAs

To verify the target lncRNAs obtained in the microarray analysis, the sample size was further expanded, and the target lncRNAs and mRNAs of the 12 patients and 12 healthy individuals were verified by qPCR. In this study, based on the analysis of chip data and the correlation with a disease, lncRNA: XLOC-005590, HNF1A-AS1, and mRNA: ECI2 were selected for follow-up verification. To correct errors in RNA concentration and transcription efficiency, β-actin was used as the internal reference gene. The steps of RNA extraction and RNA quality detection were the same as those used in microarray research.

The total RNA of 12 healthy individuals comprising the control group, 12 patients comprising the before JTTZR intervention group, and 12 patients comprising the after JTTZR intervention group were extracted using Trizol reagent, quantified using the Qubit method, and integrity checked by agarose gel electrophoresis. Both lncRNAs and mRNAs were expressed using β-actin as the internal reference gene. Real-time PCR was performed for the target gene and β-actin was used for each sample. The concentrations of target genes and β-actin in each sample were generated directly according to a plotted gradient dilution DNA standard curve. The concentration of the target gene in each sample was divided by the concentration of β-actin, to obtain the corrected relative content of this gene in the sample. Primers for the lncRNAs and mRNAs are shown in **Table 2**.

**Table 2:** The primer.

Name	Sequence (5′-3′)	Annealing temperature (°C)	Length/bp
β-actin	F:5′CCTGTACGCCAACACAGTGC3′ R:5′ATACTCCTGCTTGCTGATCC3′	60	211
XLOC-005590	F:5′CTCTGGCTCTCAATGTGTTCC3′ R:5′TCCCAGGCAATCCATTTATC3′	60	119
HNF1A-AS1	F:5′GAGTGTcCCTTCAGCCAGTC3′ R:5′TGGGAAGGAGAACAGTCCGA3′	60	116
ECl2	F:5′GCCATAAACACTGAGATGTATC3′ R:5′TGATTGAGTCATCCTTGCTG3′	60	70

### Statistical Analysis

Statistical analysis was performed using SPSS 17.0, the enumeration data usage frequency was statistically described, and the test was adopted. The measurement data were statistically described as the mean ± standard error, and a two-sided test was used. Statistical significance was set at p < 0.05. For comparison of measurement data before and after the intervention, if the differences between the data were normally distributed, the paired t-test was used; for the data whose difference did not conform to the normal distribution, the rank-sum test of the nonparametric test of two related samples was used. When comparing between groups, if the measurement data obeyed the normal distribution and met the homogeneity of variance, the two independent sample t-test was used; if the homogeneity of variance was not satisfied, the t-test was used; if the t-test conditions were not met, the rank-sum test of the two independent samples nonparametric test was used.

### Bioinformatics Analysis

The differences between the groups of lncRNAs and mRNAs were screened according to the comparison between the groups (p < 0.05 and | fold change| ≥2.0). The mean value of the multiple difference was calculated after the Ct value of each gene was detected by qPCR. The data analysis was roughly divided into the following five parts: lncRNA/mRNA differential expression analysis, Gene Ontology (GO) analysis, Pathway analysis, target lncRNAs, mRNA qPCR verification result analysis, target lncRNA differential expression, and various clinical indicator correlation analyses. The DAVID function annotation table was used for functional enrichment analysis of differentially expressed mRNAs (P<0.05) (3). According to the International Standard Classification System of Gene Function, GO is divided into three parts: molecular function, biological process, and cellular component.

## Results

### General Condition of the Patient and the Efficacy of JTTZR

The general condition of the patients in the disease and healthy control groups are shown in **Table 3**. There was no statistical difference in sex or age (p > 0.05), and they were comparable.

**Table 3:** Comparison of the general situation of the two groups.

General situation	Disease group		Healthy control group		statistic of test	Р-Value
	Male	Female	Male	Female		
Gender	7	5	6	6	0.168	0.682
Age (years old)	54.00 ± 7.79		52.92 ± 10.18		0.293	0.772

In the disease group, after 24 weeks of JTTZR intervention (**Table 4**), the changes in glucose and lipid metabolism and obesity-related clinical indicators were as follows: HbA1c decreased (1.73 ± 0.74)%, FPG decreased (1.54 ± 1.60) mmo1/L, 2hPG decreased (4.55 ± 3.62) mmol/L, TG decreased (1.18 ± 1.80) μmol/L, weight decreased (3.19 ± 2.75) kg, BMI decreased (1.23 ± 1.07) kg/m^2^, and waist circumference decreased (3.54 ± 3.29) cm. The differences were statistically significant (p < 0.05) and were therefore suitable for follow-up mechanism research.

**Table 4:** Comparison of clinical indicators before and after JTTZR intervention (n=12).

	0 week	24th week	0–24th week	Statistic of test	Р-Value
HbA1c (%)	7.82 ± 0.53	6.09 ± 0.72	1.73 ± 0.74	8.116	<0.001
FPG (mmol/L)	8.62 ± 1.60	7.08 ± 0.58	1.54 ± 1.60	3.333	0.007
2hPG (mmol/L)	15.95 ± 4.74	11.39 ± 3.31	4.55 ± 3.62	4.362	0.001
TG (mmol/L)	3.93 ± 3.82	2.75 ± 2.19	1.18 ± 1.80	-2.550	0.011
Body weight (kg)	77.99 ± 12.42	74.80 ± 12.70	3.19 ± 2.75	4.027	0.002
BMI (kg/m^2^)	28.71 ± 4.20	27.48 ± 4.03	1.23 ± 1.07	3.983	0.002
Waist circumference (cm)	97.79 ± 19.40	94.25 ± 8.74	3.54 ± 3.29	3.734	0.003
TC	5.77 ± 1.26	4.90 ± 1.15	0.87 ± 0.72	1.779	0.089
HLD-C	1.26 ± 0.36	1.05 ± 0.18	0.21 ± 0.35	1.8	0.086
LDL-C	3.41 ± 0.63	2.79 ± 0.75	0.63 ± 0.60	2.206	0.038
WBC	6.45 ± 1.07	5.93 ± 1.19	0.52 ± 0.71	1.127	0.272
RBC	4.71 ± 0.40	4.64 ± 0.48	0.08 ± 0.46	0.419	0.679
Hemoglobin	141.50 ± 11.87	140.25 ± 10.17	1.25 ± 11.75	0.277	0.784
PLT	198.58 ± 68.58	184.33 ± 71.35	14.25 ± 26.10	0.4988	0.623
ALT	33.86 ± 19.49	23.93 ± 10.18	9.93 ± 16.19	1.563	0.132
AST	24.42 ± 8.74	21.05 ± 3.87	3.37 ± 7.79	1.179	0.251
CRE	63.75 ± 7.81	62.94 ± 10.78	0.81 ± 7.83	0.2103	0.835
BUN	5.76 ± 1.40	5.11 ± 1.13	0.65 ± 1.42	1.255	0.228

### RNA Results

1. NanoDrop was used to detect the concentration of nucleic acids in the sample, the ratio of protein to nucleic acids, and whether there was contamination. The concentration of the sample and absorbance at 230, 260, and 280 nm were recorded. The 260/280 ratio reflects the ratio of the RNA concentration to the protein concentration. The ideal value should be 1.8–2.0. A ratio lower than 1.8 indicates that there is protein or other contamination. The 260/230 ratio was used to reflect the ratio of RNA concentration to coextraction contamination. The ideal value is 1.8–2.2. A ratio lower than 1.8 indicates the presence of organic matter/salt ions and other pollutants, which will affect the subsequent qPCR reaction. The RNA purity test of all samples used in the experiment showed that the absorbance ratio of A260/A280 was between 1.8–2.1, indicating that it can be used for follow-up research.
2. RNA integrity detection: The integrity of RNA was analyzed by gel electrophoresis to ensure the quality of RNA and to prevent RNA degradation from affecting subsequent experiments. The general standard is a 28S/18S ratio of >1.5(high quality), 1.3–1.5 is barely usable; and <1.3, is poor quality. The test showed that each electrophoresis band contained two bands of 28S and 18S, and the ratio of A260/A280 was between 1.8–2.1, indicating that the RNA was of good integrity and could be used for follow-up research (**Figure S1A**).
3. Comparison of lncRNA/mRNA expression levels: FPKM is the expected number of fragments per kilobase of transcript sequence per million base pairs sequenced. It also considers the impact of sequencing depth and gene length on fragment counts and is currently the most commonly used method for estimating gene expression levels. FPKM box plots of all transcripts were used to compare the expression levels under different experimental conditions. Where samples were repeated under the same experimental conditions, the final FPKM was calculated as the average of all repeated data. As shown in **Figure S1B**, the lncRNA/mRNA expression levels of each sample were similar, and the overall quality of sequencing was good.

### Gene Chip Results

The sequencing principle of the gene chip was based on hybrid sequencing. In microarray analysis, fluorescently labeled DNA bases have different absorption and emission light at different wavelengths. The probe was fluorescently labeled, and the hybridized chip was scanned (Agilent DNA Microarray Scanner) to obtain a scan (**Figure S1C**). The Arraystar human lncRNA chip V3.0 chip was selected, which can detect 30,586 lncRNAs and 26,109 protein-coding transcripts. The Agilent Feature Extraction software was used to collect the chip probe signal values. Agilent GeneSpring GX v12.1 software was used to standardize the chip and select differentially expressed lncRNAs/mRNAs. Based on the difference in the expression intensity of specific lncRNAs/mRNAs, the differentially expressed lncRNAs/mRNAs in the disease group before treatment, healthy control group, and disease group after treatment/before treatment were screened out.

### Gene Chip Data Analysis

#### Disease Group Before Treatment/Healthy Control Group

The disease group was compared with the healthy control group, and differential genes were screened according to P < 0.05 and |fold change| ≥ 2.0. A total of 557 lncRNAs were upregulated and 273 were downregulated. There were 491 differentially expressed mRNAs up-regulated and 1639 down-regulated (**Table S1**, see **Supplementary Materials**).

**Figure 2:**
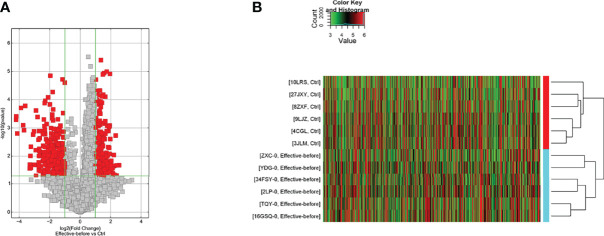
Differentially expressed lncRNA in the disease group compared to the before treatment/healthy control group [**(A)** Volcano map; **(B)** Clustering heat map].

The differences in the lncRNAs in the disease group compared to the before treatment/healthy control group were clustered in one way. The red color in the figure represents the high relative expression of lncRNAs, and the green represents the low relative expression. Each column is the sample name and each row represents a differentially expressed lncRNA. Through cluster analysis, it can be seen that the two groups of samples are grouped into two clusters, indicating that from the perspective of differences in lncRNAs, the two groups have similar biological characteristics (**Figures 2A, B**).

#### Disease Group After Treatment/Before Treatment

**Figure 3:**
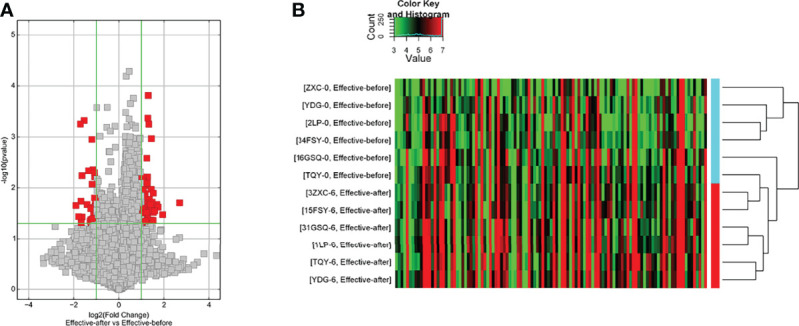
Differentially expressed lncRNA in disease group after treatment and before treatment [**(A)** Volcano map; **(B)** Clustering heat map].

The disease group after treatment was compared with the disease group before treatment, and differential genes were screened according to P < 0.05 and |fold change| ≥ 2.0. There were 128 upregulated and 32 downregulated lncRNAs and 45 upregulated and 140 downregulated mRNAs (**Table S2**). The differences in lncRNAs in the disease group after treatment and before treatment were clustered in one way. Through cluster analysis, it can be seen that the two groups of samples are grouped into two clusters, indicating that from the perspective of different lncRNAs, the two groups have similar biological characteristics (**Figure 3**).

### Bioinformatics Analysis of Differentially Expressed mRNA

#### Disease Group Before Treatment/Healthy Control Group

(1) Upregulated mRNA analysis

**Figure 4:**
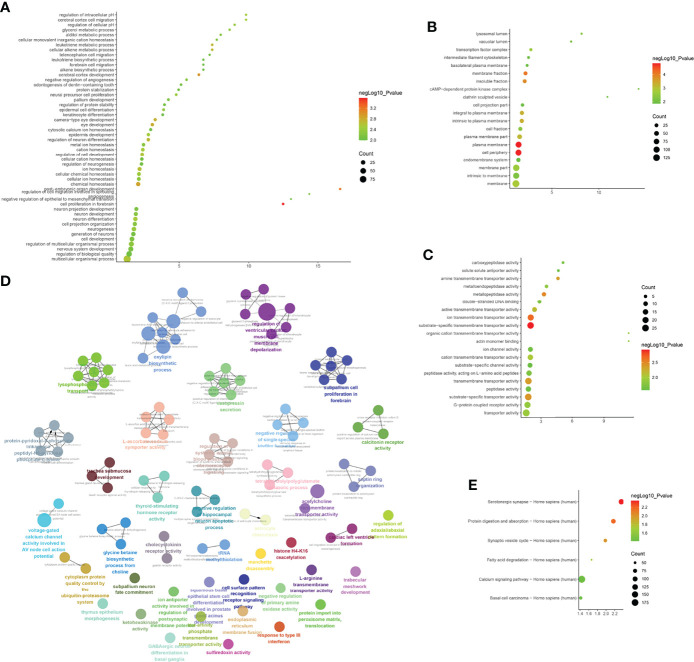
Enrichment Analysis of up-regulated mRNA [**(A)** biological processes; **(B)** cell components; **(C)** molecular functions; X-axis stands for fold enrichment. **(D)** enrichment results; **(E)** signaling pathways; X-axis stands for enrichment score.

The biological processes identified included cell proliferation in the forebrain, post-embryonic organ development, cerebral cortex development, chemical homeostasis, eye development, camera-type eye development, leukotriene metabolic processes, cellular alkene metabolic processes, regulation of neuron differentiation, and ion homeostasis. The cell components identified included the plasma membrane, cell periphery, membrane fraction, insoluble fraction, intrinsic to the plasma membrane, integral to the plasma membrane, plasma membrane part, membrane transcription factor complex, and membrane part. Molecular functions identified include substrate-specific transmembrane transporter activity, ion transmembrane transporter activity, metallopeptidase activity, substrate-specific transporter activity, transmembrane transporter activity, amine transmembrane transporter activity, active transmembrane transporter activity, cation transmembrane transporter activity, transporter activity, and actin monomer binding. The signaling pathways identified included serotonergic synapses, protein digestion and absorption, the synaptic vesicle cycle, fatty acid degradation, the calcium signaling pathway, and basal cell carcinoma (**Figure 4**). The details of all enrichment results are shown in **Table S3**.

(2) Downregulated mRNA analysis

**Figure 5:**
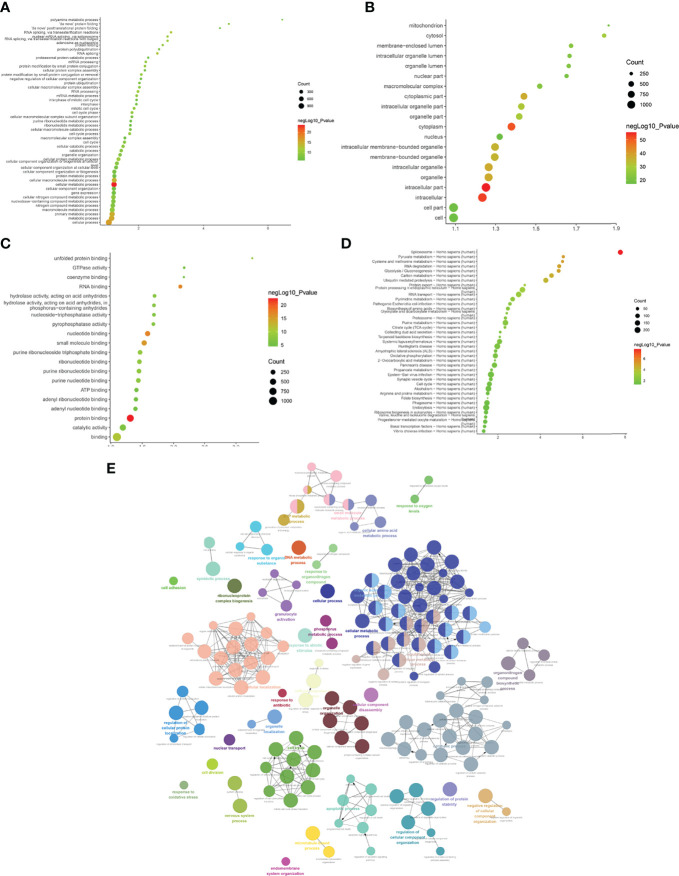
Enrichment Analysis of down-regulated mRNAs [**(A)** biological processes; **(B)** cell components; **(C)** molecular functions; X-axis stands for fold enrichment. **(D)** Signaling pathways; X-axis stands for enrichment score. **(E)** Enrichment results].

**Figure 6:**
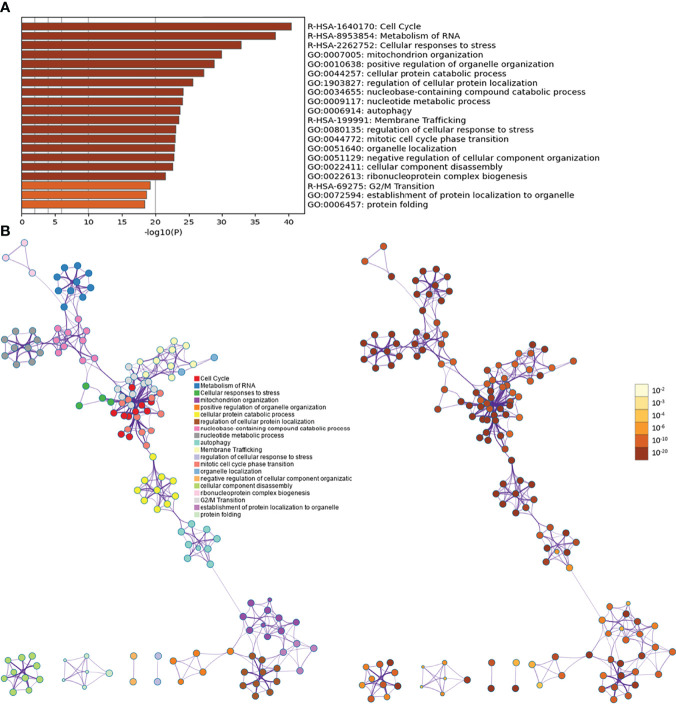
Metascape results [**(A)** Heatmap of top enrichment results; **(B)** PPI network colored by cluster and by *P*-value].

Biological processes associated with downregulated mRNA expression included cellular metabolic processes, cellular processes, metabolic processes, primary metabolic processes, cellular macromolecule metabolic processes, RNA splicing, RNA splicing *via* transesterification reactions, RNA processing, splicing *via* transesterification reactions with bulged adenosine as a nucleophile, and nuclear mRNA splicing *via* spliceosome. Cell components associated with downregulated mRNA expression included intracellular part, intracellular, cytoplasm, intracellular organelle, organelle, cytoplasmic part, intracellular membrane-bounded organelle, membrane-bounded organelle, intracellular organelle part, and organelle part. Molecular functions included protein binding, nucleotide binding, RNA binding, small molecule binding, binding, unfolded protein binding, purine ribonucleoside triphosphate binding, ribonucleotide binding, purine ribonucleotide binding, and purine nucleotide binding. The signaling pathways included spliceosome, pyruvate metabolism, cysteine and methionine metabolism, RNA degradation, glycolysis/gluconeogenesis, carbon metabolism, ubiquitin-mediated proteolysis, protein export, protein processing in the endoplasmic reticulum, and RNA transport (**Figure 5**). The details of all enrichment results are shown in **Table S4**. All differentially expressed mRNAs were imported into Metascape for visualization (**Figure 6**).

#### Disease Group After Treatment/Before Treatment

(1) Upregulated mRNA analysis

**Figure 7:**
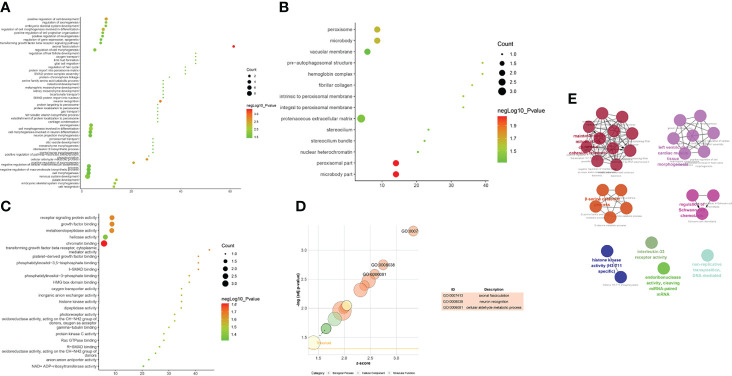
Enrichment Analysis of up-regulated mRNAs [**(A)** biological processes; **(B)** cell components; **(C)** molecular functions; X-axis stands for fold enrichment. **(D)** go bubble chart of top GO results; **(E)** enrichment results].

The biological processes associated with upregulated mRNA expression included axonal fasciculation, neuron recognition, cellular aldehyde metabolic processes, positive regulation of cell development, positive regulation of axonogenesis, regulation of cell morphogenesis involved in differentiation, palate development, embryonic skeletal system morphogenesis, cell recognition, and cell morphogenesis involved in differentiation. The cell components associated with upregulated mRNA expression included microbody part, peroxisomal part, peroxisome, microbody, pre-autophagosomal structure, hemoglobin complex, fibrillar collagen, integral to the peroxisomal membrane, intrinsic to the peroxisomal membrane, and proteinaceous extracellular matrix. Molecular functions included chromatin binding, transforming growth factor-beta receptor, cytoplasmic mediator activity, receptor signaling protein activity, growth factor binding, metalloendopeptidase activity, platelet-derived growth factor binding, I-SMAD binding, phosphatidylinositol-3,5-bisphosphate binding, phosphatidylinositol-3-phosphate binding, and HMG box domain binding. These signaling pathways included peroxisomes (**Figure 7**). The details of all the enrichment results are presented in **Table S5**.

(2) Downregulated mRNA analysis

**Figure 8:**
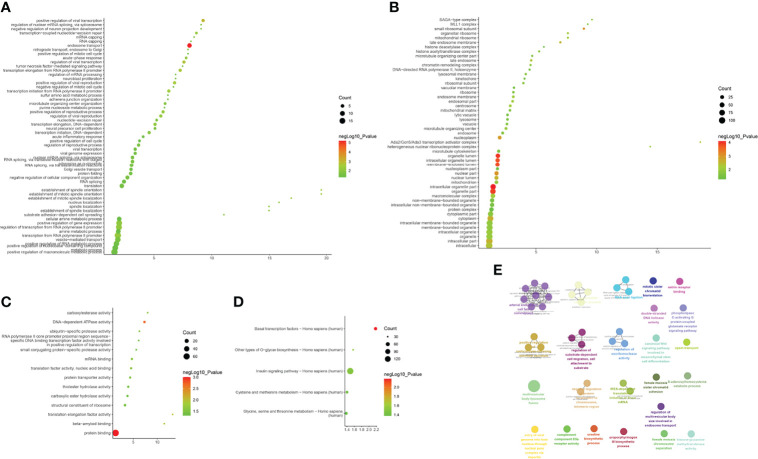
Enrichment Analysis of down-regulated mRNAs [**(A)** biological processes; **(B)** cell components; **(C)** molecular functions; X-axis stands for fold enrichment. **(D)** Signaling pathway; X-axis stands for enrichment score. **(E)** Enrichment results].

**Figure 9:**
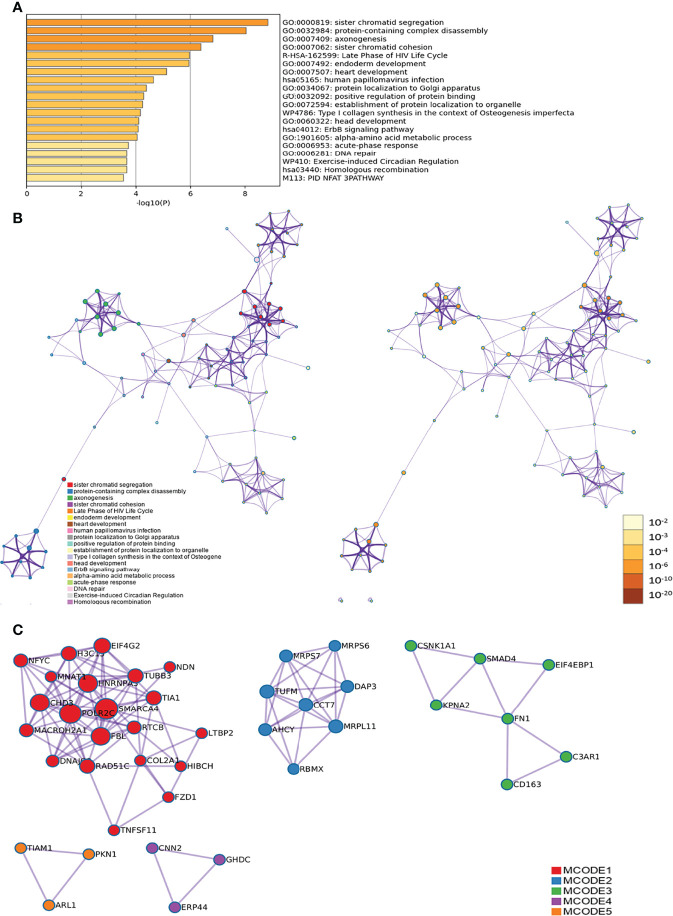
Metascape results [**(A)** Heatmap of top enrichment results; **(B)** PPI network colored by cluster and **(C)** PPI network colored by *P*-value].

Biological processes associated with downregulated mRNA expression included endosome transport, positive regulation of viral transcription, regulation of viral transcription, transcription elongation from RNA polymerase II promoter, positive regulation of viral reproduction, transcription initiation from RNA polymerase II promoter, the establishment of mitotic spindle orientation, the establishment of spindle orientation, transcription-coupled nucleotide-excision repair, and positive regulation of reproductive processes. The cell components included associated with upregulated mRNA expression the intracellular organelle part, organelle lumen, organelle part, membrane-enclosed lumen, intracellular organelle lumen, nuclear part, small ribosomal subunit, nucleoplasm, cytoplasm, and intracellular part. Molecular functions included protein binding, DNA-dependent ATPase activity, translation elongation factor activity, beta-amyloid binding, carboxylesterase activity, mRNA binding, translation factor activity, nucleic acid binding, protein transporter activity, structural constituents of ribosomes, and ubiquitin-specific protease activity. The signaling pathways included basal transcription factors, other types of o-glycan biosynthesis, the insulin signaling pathway, cysteine and methionine metabolism, and glycine, serine, and threonine metabolism (**Figure 8**). The details of all the enrichment results are presented in **Table S6**. All differentially expressed mRNAs were imported into Metascpe for visualization (**Figure 9**).

### Bioinformatics Analysis of Differentially Expressed LncRNA

#### Disease Group Before Treatment/Healthy Control Group

**Figure 10:**
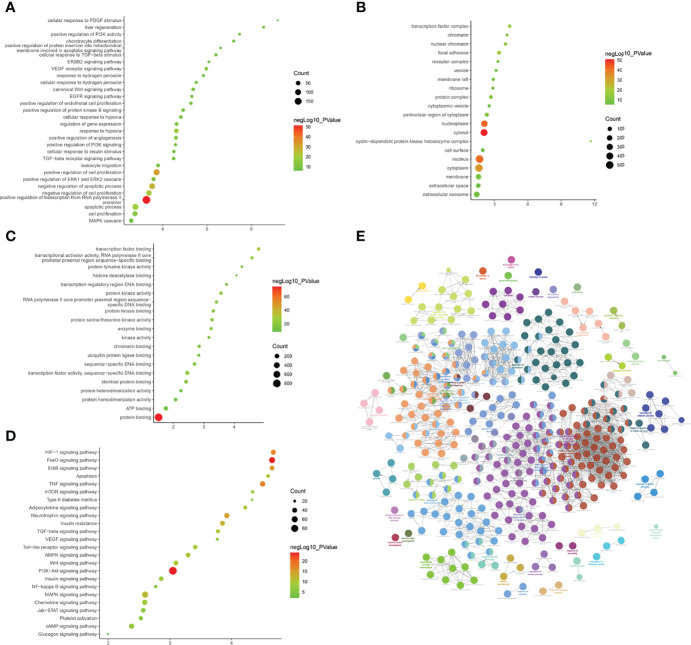
Enrichment Analysis of lncRNA [**(A)** biological processes; **(B)** cell components; **(C)** molecular functions; X-axis stands for fold enrichment. **(D)** Signaling pathway; X-axis stands for enrichment score. **(E)** Enrichment results].

LncACTdb (http://www.bio-bigdata.net/LncACTdb/) was used to predict mRNA corresponding to the lncRNA, following which the mRNA sequences were imported into DAVID for enrichment analysis. The biological processes identified included positive regulation of transcription from the RNA polymerase II promoter, positive regulation of cell proliferation, negative regulation of the apoptotic process, negative regulation of cell proliferation, apoptotic process, response to hypoxia, VEGF receptor signaling pathway, positive regulation of angiogenesis, cell proliferation, canonical Wnt signaling pathway, and cellular response to hypoxia. The cell components identified included the cytosol, nucleoplasm, nucleus, cytoplasm, focal adhesion, transcription factor complex, nuclear chromatin, protein complex, perinuclear region of cytoplasm, and membrane. Molecular functions included protein binding, transcription factor binding, transcriptional activator activity, RNA polymerase II core promoter proximal region sequence-specific binding, transcription factor activity, sequence-specific DNA binding, protein kinase activity, RNA polymerase II core promoter proximal region sequence-specific DNA binding, protein kinase binding, protein serine/threonine kinase activity, identical protein binding, enzyme binding, sequence-specific DNA binding, and transcription regulatory region DNA binding. The signaling pathways identified included the PI3K-Akt signaling pathway, FoxO signaling pathway, TNF signaling pathway, HIF-1 signaling pathway, ErbB signaling pathway, neurotrophin signaling pathway, insulin resistance, MAPK signaling pathway, apoptosis, AMPK signaling pathway, adipocytokine signaling pathway, Wnt signaling pathway, TGF-β signaling pathway, and Toll-like receptor signaling pathway (**Figure 10** and **Table S7**).

#### Disease Group After Treatment/Before Treatment

**Figure 11:**
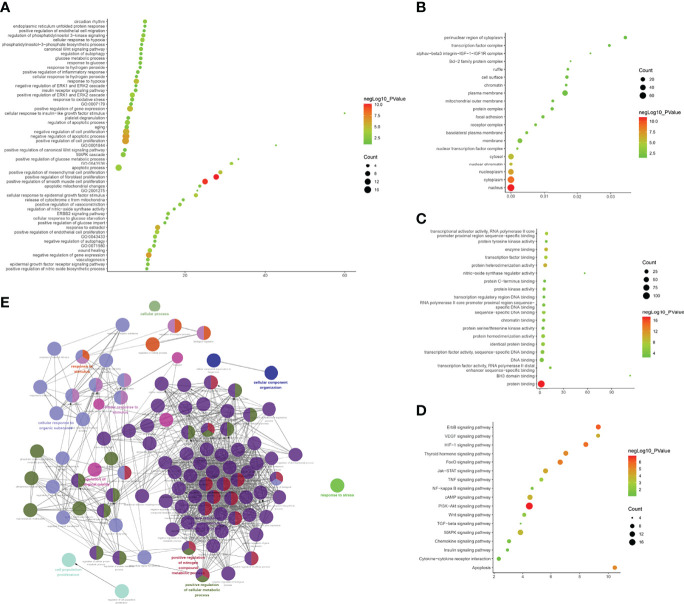
Enrichment Analysis of lncRNA [**(A)** biological processes; **(B)** cell components; **(C)** molecular functions; X-axis stands for fold enrichment. **(D)** Signaling pathway; X-axis stands for enrichment score. **(E)** Enrichment results].

The biological processes included positive regulation of fibroblast proliferation, positive regulation of smooth muscle cell proliferation, negative regulation of gene expression, negative regulation of the apoptotic process, positive regulation of cell proliferation, negative regulation of cell proliferation, response to estradiol, positive regulation of gene expression, response to hypoxia, positive regulation of mesenchymal cell proliferation, cellular response to epidermal growth factor stimulus, positive regulation of endothelial cell proliferation, and positive regulation of the ERK1 and ERK2 cascade. Cell components included the nucleus, cytoplasm, nucleoplasm, nuclear chromatin, cytosol, nuclear transcription factor complex, membrane, basolateral plasma membrane, and receptor complex. Molecular functions included protein binding, protein heterodimerization activity, enzyme binding, transcription factor binding, transcriptional activator activity, RNA polymerase II core promoter proximal region sequence-specific binding, protein homodimerization activity, sequence-specific DNA binding, identical protein binding, transcription factor activity, sequence-specific DNA binding, and protein kinase activity. The signaling pathways included the PI3K-Akt, ErbB, HIF-1, FoxO, apoptosis, thyroid hormone, Jak-STAT, VEGF, and cAMP signaling pathways (**Figure 11** and **Table S8**).

### lncRNA-mRNA Co-Expression Network Analysis

**Figure 12:**
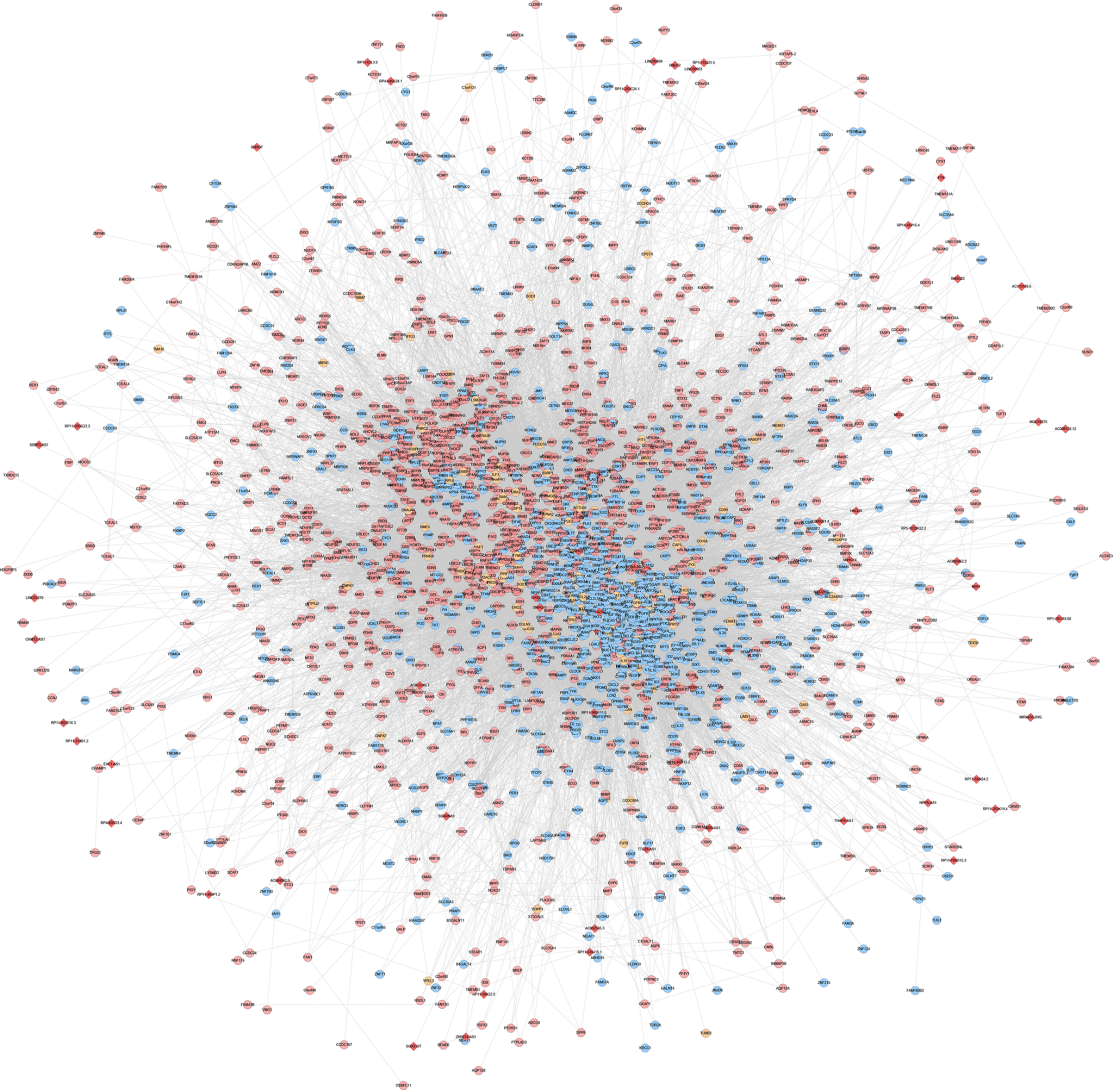
LncRNA-mRNA co-expression network of the disease group before treatment/healthy control group (Red diamonds represent lncRNA. Blue, pink, and orange circles represent mRNA predicted by the lncRNA, mRNA detected by the experiment and mRNA that interacts with lncRNA, respectively).

**Figure 13:**
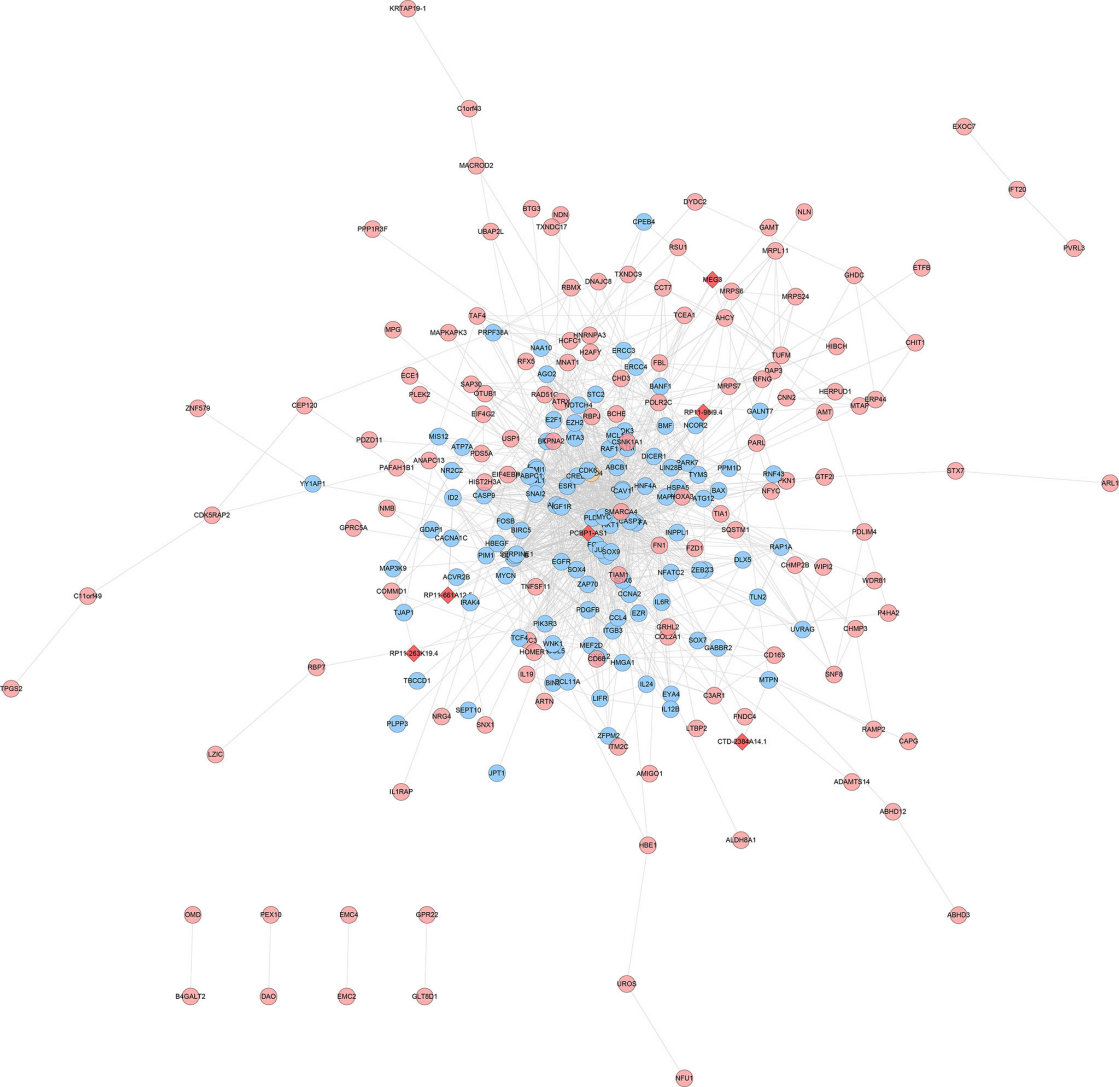
LncRNA-mRNA co-expression network of the disease group after treatment/before treatment (Red diamonds represent lncRNA. Blue, pink, and orange circles represent mRNA predicted by the lncRNA, mRNA detected by the experiment and mRNA that interacts with lncRNA, respectively).

The experimentally detected mRNA, lncRNA-predicted mRNA, and the interaction relationship between mRNA and lncRNA were imported into Cytoscape for analysis. It was found that in the co-expression network, there is an interaction between the mRNA detected by the experiment and the mRNA predicted by the lncRNA, and some of the mRNA detected by the experiment directly interact with the lncRNA (orange circles in the network) (**Figures 12**, **13**).

### qPCR Verification of Target lncRNAs and mRNAs

According to the systematic inquiry and comparison of the expression differences of gene chip lncRNAs and mRNAs, the analysis of GO and Pathway enrichment information, and the correlation with disease, this study verified the expression of two lncRNAs (XLOC-005590, HNF1A-AS1) and their related mRNA (ECI2) by qPCR.

The results of gene chip sequencing showed that the expression of XLOC-005590 and HNF1A-AS1 was upregulated in obese T2DM patients with dyslipidemia compared with that in healthy volunteers, and the downstream ECI2 expression was significantly downregulated. After intervention with JTTZR for 24 weeks, the expression of XLOC-005590 was downregulated and the expression of ECI2 was upregulated. (**Table 5**)

**Table 5:** Expression of XLOC_005590, HNF1A-AS1 and ECI2 in gene chip.

Group	Gene symbol	*P*-value	Log2FC	Regulation	Genome relationship
Disease group before treatment/healthy control group	XLOC_005590	0.025272	2.330217	up	/
	HNF1A-AS1	0.002463	3.897015	up	/
Disease group after treatment/before treatment	ECI2	0.004332	-3.630339	down	downstream
	XLOC_005590	0.047567	-2.336840	down	/
	HNF1A-AS1	0.002463	3.897015	up	/
	ECI2	0.752882	1.116477	up	downstream

**Figure 14:**
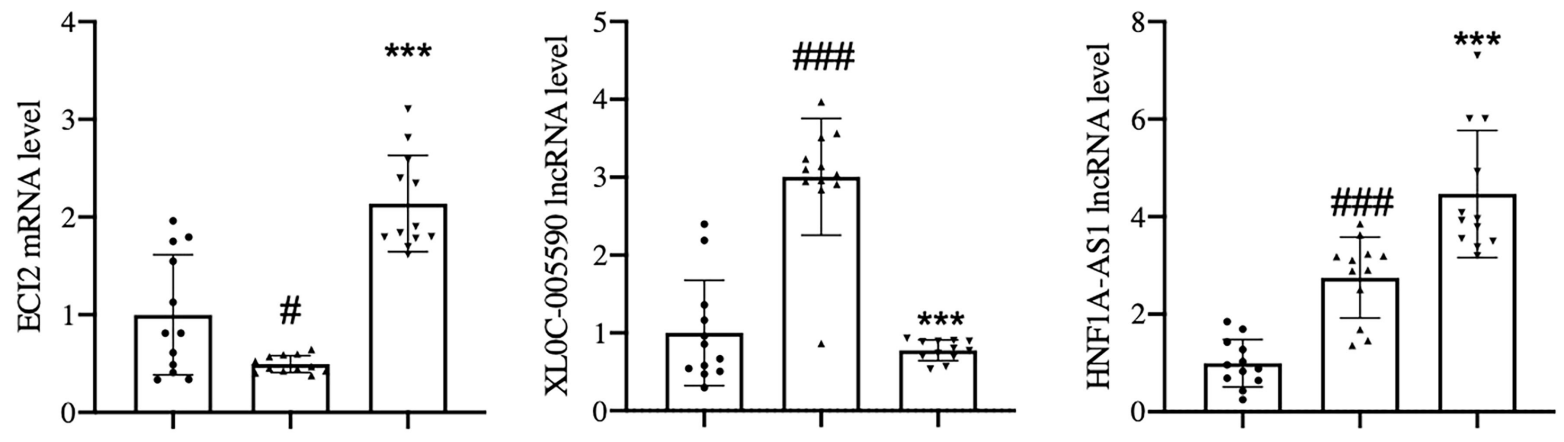
qPCR was used to verify the expression of XLOC_005590 and ECI2 in the healthy control group, and the disease group before and after treatment. The left column represents the healthy control group, the middle column represents the disease group before treatment, and the right column represents the disease group after treatment. The normalization using β-actin. ^#^P < 0.05, ^###^P<0.001, versus the healthy control group. ***P < 0.001, versus the before treatment group; n= 12.

Therefore, the sample size was expanded to 12 cases in each group, and qPCR verification was performed. The results showed that with reference to the healthy control group, the expression of the target lncRNA (XLOC-005590, HNF1A-AS1) and its related mRNA (ECI2) showed the same trend as the gene chip results. After 24 weeks of JTTZR intervention, a comparison with the pre-treatment group (week 0), revealed that the expression of XLOC_005590 was significantly downregulated, whereas the expression of downstream ECI2 was upregulated, and the difference was statistically significant (P < 0.05). (**Figure 14**)

### Correlation Analysis Between Target lncRNAs and Clinical Indicators

Correlation analysis was performed between the relative expression of target lncRNAs in the qPCR results and the clinical indicators of sugar, lipid, and fat in obese T2DM patients with dyslipidemia to understand the relationship between the expression of XLOC-005590 and HNF1A-AS1 and disease.

**Figure 15:**
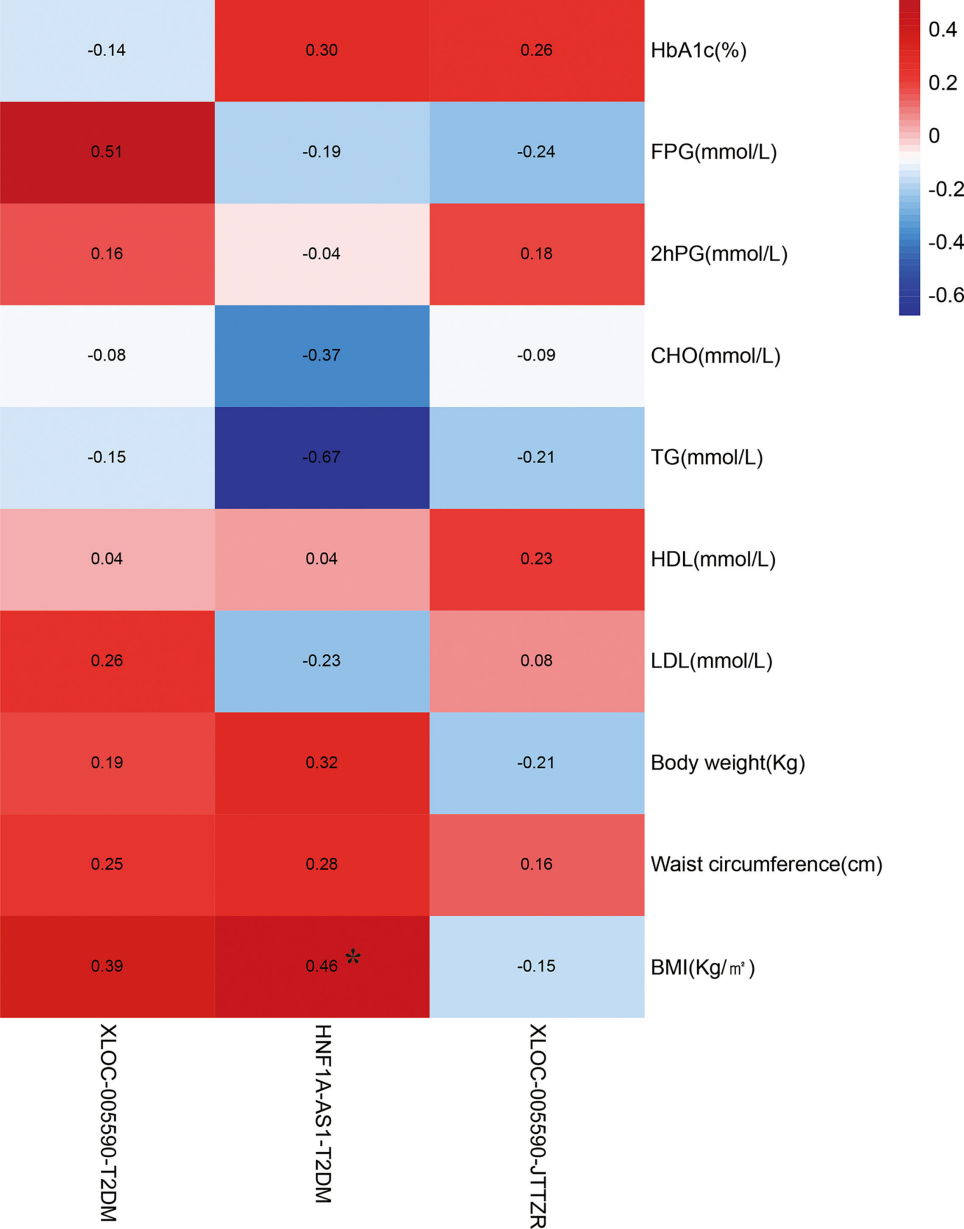
Correlation analysis between changes in clinical indicators and target lncRNA in the qPCR results. *P < 0.05.

The correlation analysis between the relative expression of HNF1A-AS1 and BMI of obese T2DM patients with dyslipidemia suggested that the correlation coefficient of the two variables was 0.455, P=0.040 (<0.05). The relative expression of HNF1A-AS1 can be considered to have a positive linear correlation with the BMI of obese T2DM patients with dyslipidemia. The correlation analysis between the relative expression of XLOC-005590 and clinical indicators suggests that the *P*-value of each group’s correlation analysis is > 0.05, and it cannot, therefore, be conclusively stated that the relative expression difference of XLOC-005590 is positively linearly related to the difference in the clinical indicators of glucose, lipid, and fat in obese T2DM patients with dyslipidemia (**Figure 15**).

## Discussion

Our previous studies (NCT04623567) have confirmed that JTTZR can significantly reduce the components of metabolic syndrome and cardiovascular risk factors in obese T2DM patients with dyslipidemia (*excessive gastrointestinal heat syndrome*), such as glycosylated hemoglobin, blood sugar, triglyceride, total cholesterol, high-density lipoprotein cholesterol, waist circumference, hip circumference, and body weight. It can also improve the function of pancreatic β-cells and insulin resistance (2). The effects of JTTZR were similar to those of metformin. It is superior to metformin in improving TCM syndromes, which preliminarily illustrates the effectiveness of JTTZR on the homology of glucose, lipids, and fat in patients with T2DM. Based on the above results, this study further explored the mechanism of regulation of the lncRNA-mRNA molecular biological network of obese T2DM patients with dyslipidemia (*excessive gastrointestinal heat syndrome*). The results of pathway enrichment analysis of differential genes in the disease group before treatment and healthy people showed that there are six significant signal transduction pathways involved in the upregulation of differential mRNAs, such as fatty acid degradation, and 39 significant signal transduction pathways involved in the downregulation of differential mRNAs, such as glycolysis/gluconeogenesis and pyruvate metabolism. The expression comparison of these pathways reflects the characteristic differences between obese T2DM patients with dyslipidemia and healthy individuals.

In the KEGG pathway analysis, the fatty acid degradation pathway is a subordinate item of the metabolic process-lipid metabolism. Fatty acid degradation refers to the process by which fatty acids are broken down into their metabolites and acetyl-CoA is produced, which enters the citric acid cycle and becomes the body’s main energy supply. It consists of three main steps: lipolysis and release from adipose tissue, activation and transport to the mitochondria, and β-oxidation. Fatty acids are initially stored in fat cells. After the fat is broken down, free fatty acids (FFAs) are released into the blood and participate in the circulation of the whole body. After fatty acids are activated to acyl-CoA, they are taken into the mitochondria to initiate β-oxidation. The differentially expressed acyl-CoA synthetase long-chain family member (4ACSL4) was identified in this study is an important part of this pathway. Fatty acids require acyl-CoA synthetase to activate acyl-CoA. In addition, some medium and long-chain fatty acids and a small amount of long-chain fatty acids participate in the omega-oxidation process, and after a series of steps, dicarboxylic acids are generated and then beta-oxidized. The A1aldehyde dehydrogenase 7 family, member A1 (ALDH7) and cytochrome P450, family 4, subfamily A, polypeptide 11 (CYP4A11) are related to this special oxidation process. In obese T2DM patients with dyslipidemia, increased body fat storage can lead to the upregulation of fatty acid degradation pathways. Plasma FFA levels are significantly increased, and through the classic glucose-fatty acid cycle pathway, insulin-mediated glucose uptake and utilization of glucose metabolism organs, such as the liver and muscle, are reduced. Increased FFA levels can also further damage the function of pancreatic β-cells through “lipotoxicity”.

Compared with healthy people, the upregulation of the fatty acid degradation pathway reflects the characteristic pathological changes in obese T2DM patients with dyslipidemia. The fatty acid degradation pathway is closely related to diabetes, and the fatty acid β-oxidation pathway of eukaryotic cells mainly exists in two organelles, peroxisomes and mitochondria. In diabetic patients, due to the relative or absolute lack of insulin, the body is more dependent on fat for energy, and mitochondrial fatty acid beta oxidation activity increases. In addition, several studies have shown that the β-oxidation activity of peroxisomal fatty acids is enhanced in diabetes (4–8). In summary, studies have shown that fatty acid β-oxidation activity is increased in patients with diabetes, which is consistent with the upregulation of fatty acid degradation pathway enrichment in the disease group after treatment/before treatment in this study.

Fatty acid degradation pathway and metabolic syndrome: Diabetes patients with metabolic syndrome have disorders of adiponectin and free fatty acid metabolism. Studies have suggested that adiponectin levels decrease, and total fatty acids and polyunsaturated fatty acids increase, which may be the basis or risk factors for the onset of diabetes and metabolic syndrome. TCM-related research shows that (1) *Radix Rhei Et Rhizome* interferes with fatty acid degradation: Rhein can reduce plasma FFA, thereby improving insulin resistance (9). *Radix Rhei Et Rhizome* alcohol extract can improve insulin sensitivity in diabetic obese rats and reduce plasma free fatty acid and blood lipid levels (10). *Radix Rhei Et Rhizome* extract tablets showed a dose-dependent enhancement of lipid-lowering effects, and intervention in obese rats induced by a high-fat diet has a weight loss effect (11). (2) *Coptidis Rhizoma* interferes with FFA degradation: Relevant animal experiments show that *Coptidis Rhizoma* reduces saturated fatty acids and increases monounsaturated fatty acids and polyunsaturated fatty acids compared to model mice, similar to those in normal mice, which suggests that *Coptidis Rhizoma* decoction has a better regulation and treatment effect on fatty acid abnormalities in diabetes (12). JTTZR contains *Radix Rhei et Rhizoma* and *Coptidis Rhizoma*. The possible mechanism whereby this prescription interferes with obese T2DM with dyslipidemia is that *Radix Rhei Et Rhizom* and *Coptidis Rhizoma* act by regulating the fatty acid degradation pathways.

In the KEGG pathway analysis, downregulation of the glycolysis/gluconeogenesis pathway is a subordinate item of metabolic process-carbohydrate metabolism. Glycolysis is the conversion of glucose to pyruvate and the production of small amounts of ATP and NADH. Glycolysis provides energy for cell metabolism and is one of the main routes for blood sugar production. Downregulation of the glycolytic pathway can lead to hyperglycemia, which is consistent with the pathological state of obese T2DM combined with dyslipidemia described in this study. Excluding some minor changes in pathways, gluconeogenesis can be considered as the reverse reaction of glycolysi7s, that is, the re-synthesis of glucose or glycogen from non-sugar compounds, such as pyruvate, glycerol, and lactic acid. Hepatic gluconeogenesis disorder is closely related to insulin resistance diseases such as diabetes, obesity, and non-alcoholic fatty liver disease. It effectively inhibits excessive gluconeogenesis in the liver and reduces endogenous glucose production, which is one of the important targets for the treatment of T2DM. For example, berberine can reduce excessive hepatic glucose production (HGP) by limiting the mitochondrial import of pyruvate through MPC1, which is an important mechanism for the treatment of T2DM (13). Some intermediate products of glycolysis/gluconeogenesis are synthetic precursors of lipids and amino acids that are closely related to other metabolic pathways. The signal transduction pathway that was significantly downregulated at the same time as glycolysis was pyruvate metabolism. Patients with T2DM are characterized by disorders of glucose and lipid metabolism, inhibition of glycolysis and aerobic oxidation processes, and enhancement of gluconeogenesis. Therefore, this study focused on issues related to the downregulation of glycolysis. Glycolysis is closely related to abnormal glucose and lipid metabolism. Key enzymes (glucokinase, phosphofructokinase, and pyruvate kinase) are indispensable for the reaction process in the glycolysis pathway (14–16). Relevant studies on TCM intervention in glycolysis have shown that Jiaotai pills, *Coptidis rhizoma*, and *Cinnanmomi cortex* have significant effects on plasma metabolic components in T2DM rats. By analyzing the differences in its metabolic components, it is speculated that *Coptidis Rhizoma* may affect certain links of the tricarboxylic acid cycle and promote the aerobic oxidation of glucose, whereas *Cinnanmomi Cortex* mainly promotes the anaerobic glycolysis of sugar. *Coptidis Rhizoma* and *Cinnanmomi Cortex* are compatible with Jiaotai pills, which can regulate aerobic oxidation and glycolysis and inhibit the gluconeogenesis pathway. In patients with T2DM, Jiaotai pills can effectively regulate glucose and lipid metabolism. This mechanism may be achieved by promoting the aerobic oxidation of sugar and inhibiting the two metabolic pathways of gluconeogenesis, which has little effect on the glycolysis pathway (17).

The target lncRNAs and mRNAs were verified by qPCR, and the results showed that the expression analysis of XLOC-005590 (lncRNA) and ECI2 (mRNA) had the same orientation as the microarray results. HNF1A-AS1 (HNF1A antisense RNA 1, HNF1A antisense RNA 1), is a transcription factor that regulates a large number of specific genes in the liver and β cells. HNF1A (mRNA) is believed to be related to glucose, carbohydrate, and lipid metabolism. Maturity-onset diabetes of the young (MODY) is a group of autosomal dominant monogenic diseases with different clinical and genetic manifestations. Mutations in the HNFIA gene can lead to MODY, which manifests as severe β-cell dysfunction (18). Studies have shown that 38% of women with MODY3 (HNFIA) gene mutations have a history of gestational diabetes (19); HNF1A gene mutations are often seen in women with gestational diabetes (20), which shows that HNF1A plays an important role in the susceptibility to gestational diabetes. Studies have verified the expression of HNF1A-AS1 and HNF1A in pancreatic islet tissues, confirming the tissue specificity of lncRNAs (21). In this study, compared with healthy individuals, HNF1A-AS1 was significantly upregulated in the peripheral blood of patients, and the chip and qPCR verification results produced the same results. In addition, the correlation analysis between the relative expression of HNF1A-AS1 and clinical indicators suggests that the relative expression of HNF1A-AS1 is positively correlated with the BMI of obese T2DM patients with dyslipidemia, and the mechanism remains to be further studied. In summary, we speculate that HNF1A-AS1 is a disease biomarker for obese T2DM patients with dyslipidemia.

Pathway analysis of differentially expressed genes in the disease group after treatment/before treatment showed that in the comparison of the disease group before and after treatment, there was one significant signal transduction pathway involved in upregulated differentially expressed mRNAs, which is the peroxisome. There are five significant signal transduction pathways involved in the downregulation of differentially expressed mRNAs, including the insulin signaling pathway. The expression comparison of these pathways may be closely related to the mechanism of JTTZR intervention in obesity, T2DM, and dyslipidemia. In the KEGG pathway analysis, the peroxisome pathway is a subordinate component of cellular transportation and metabolism. Peroxisomes are essential organelles that play key roles in redox signaling and lipid homeostasis. They contribute to many important metabolic processes such as fatty acid oxidation, ether lipids, and free radical scavenging biosynthesis. Since peroxisomes are involved in biological processes such as fatty acid β-oxidation, they are closely related to diseases such as abnormal glucose and lipid metabolism and obesity. The study of peroxisome proliferator-activated receptors has become a hot topic in the study of drug targets for metabolic disorders, such as diabetes. Fibrate drugs affect body weight by regulating fatty acid metabolism and energy consumption *via* PPARα. Thiazolidinediones (TZDs), developed using scanning rodent models of insulin resistance, are diabetes treatment drugs that target PPARγ. TZD series such as rosiglitazone, pioglitazone, enlitazone, and ciglitazone are PPAR agonists, which can induce fat differentiation and the expression of related lipogenes (22). Experiments on mice and non-human primates have shown that PPARδ agonists can normalize blood lipids (23–29) and reduce insulin resistance and obesity (30). PPARδ activation reduces insulin resistance by regulating mitochondrial oxidation capacity in skeletal muscle (25), and the glucose-insulin cycle, thus enhancing glucose utilization and reducing triacylglycerol deposition (26). This can reduce the level of insulin in blood circulation, increase the sensitivity of insulin to glucose, and improve glucose tolerance (28). In this study, peroxisome biosynthesis factor 10 (PEX10) in the peroxisome pathway was significantly upregulated. The protein encoded by this gene is located in the peroxisome membrane and participates in the import of peroxisome matrix proteins. Therefore, this study suggests that the mechanism of JTTZR intervention in obese T2DM with dyslipidemia may be related to the upregulation of PEX10, which enhances peroxisome biosynthesis.

In the KEGG pathway analysis, the insulin signal transduction pathway is a subitem of cellular processes, transportation, and metabolism. After the combination of insulin and insulin receptors, the signal is transmitted down to the effector mainly through three pathways, one of which is the IRS/PI3K/protein kinase B (Akt) pathway, which promotes glycogen synthesis, inhibits glycogen decomposition, and promotes glucose transporter (GLUTs) expression, ultimately regulating sugar, fat, and protein metabolism. The other is the Cap-Cbl-Crk pathway, which mainly promotes the translocation of GLUTs to the cell membrane and promotes the uptake of glucose by cells. These two pathways are metabolic signaling pathways. There is also a pathway that regulates gene transcription and cell proliferation *via* Shc/Raf/MAPK, which is the growth signaling pathway. The occurrence of T2DM combined with osteoporosis may be related to inhibition of the liver insulin signaling pathway (31). After the intervention of exogenous insulin in diabetic mice induced by a high-fat diet, the expression of Akt-2 and Akt-3 was upregulated, the expression of PIK3R2 and PTPN was downregulated, and the liver IRS/PI3K/protein kinase B (Akt) pathway was improved (32). In terms of TCM intervention in the insulin signaling pathway, studies have shown *Eugenia caryophyllata* Thunb. significantly increased the phosphorylation levels of key proteins in the insulin signaling pathway in type 2 diabetes and had a significant inhibitory effect on SHP-1. It is speculated that *Eugenia caryophyllata* Thunb. can reduce blood sugar. The mechanism involves the inhibition of the activity of the negative regulatory enzyme SHP-1 in the insulin signaling pathway, thereby enhancing insulin receptor sensitivity (33). The Chinese medicinal compound Jiangtang Yishen decoction can protect the kidneys of MKR type 2 diabetic mice by inhibiting the insulin signaling pathway in the kidney tissue. Banxia Houpu decoction is believed to restore glucose intolerance in CUMS rats by improving insulin signaling and suppressing NLRP3 inflammasome activation in the liver and brain (34). In this study, the four downregulated mRNAs that decreased in the insulin signaling pathway were EIF4, EBP1, EXOC7, PPP1R3F, and SHC3. SHC3 is the MAPK pathway adaptor protein in the Shc/Raf/MAPK pathway that regulates gene transcription and cell proliferation. Signal transduction in the MAPK pathway is mainly conducted through the RAS-MEK-MAPK cascade. RAS activation starts with the phosphorylation of the adaptor protein, SHC, by the insulin receptor substrate. Studies have shown that the metabolic regulation signaling pathway of insulin in obesity and T2DM is blocked; however, the signaling pathway that promotes proliferation is not blocked, which may lead to atherosclerosis and various microvascular complications (32). Downregulation of *Shc3* expression suggests that JTTZR does not enhance the transduction of the insulin mitogenic pathway.

The results of qPCR verification of target lncRNAs and mRNAs showed that XLOC-005590 (lncRNA) and ECI2 (mRNA):XLOC-005590 belong to a subclass of lncRNA, lncRNA (intergenic non-coding RNA). ECI2 is located downstream of XLOC-005590, and its protein is a key mitochondrial enzyme involved in the β-oxidation of unsaturated fatty acids. Based on the comparison between the two groups, it can be speculated that XLOC-005590 reversely regulates the expression of ECI2, thereby affecting glucose and lipid metabolism. The mechanism may be (1) interference with downstream gene expression through transcription in the upstream promoter region of the protein-coding gene, or (2) effect on downstream gene expression by inhibition of RNA polymerase II or mediating chromatin remodeling and histone modification. XLOC-005590 was highly expressed in 12 patients before treatment, and its expression in the disease group was significantly lower after treatment. It can be speculated that JTTZR plays a role in regulating the expression of XLOC-005590. By searching the KEGG database, we found that ECI2 is involved in the peroxisome and fatty acid degradation pathways. In the disease group after treatment/before treatment, the peroxisome pathway was significantly upregulated, and the expression of ECI2 was also upregulated compared to that before treatment, and the two showed the same trend. Studies have shown that ECI2 exists in the mitochondria and peroxisomes of mammals, is localized in the peroxisome matrix, and has an indispensable effect on the complete oxidation of unsaturated fatty acids (35). In summary, it can be inferred that XLOC-005590 regulates the expression of ECI2, and ECI2 plays a role in the peroxisome and fatty acid degradation pathways. This may be one of the mechanisms by which JTTZR interferes with obesity, T2DM, and dyslipidemia.

The advantages of this study are as follows: from the perspective of differences in lncRNA-mRNA expression, this is the first study to preliminarily explore the multi-system molecular biological network regulation mechanism of JTTZR intervention in obesity-related T2DM with dyslipidemia. The limitations are that although lncRNAs have become a research hotspot in the pathogenesis of various diseases, there are still many factors that remain unclear, and research on diseases such as diabetes, dyslipidemia, and obesity are in their infancy, and there is very little information for reference. Future research will continue to deepen our understanding of the differential lncRNAs obtained. With the continuous reports of lncRNA research, the mechanism of the obtained lncRNAs in the biological process of obese T2DM with dyslipidemia will be explored. This study included a limited population (patients with obese T2DM and dyslipidemia) and only explored the therapeutic role of JTTZR. In addition, lncRNAs (XLOC-005590) still lack animal-level verification as well as in-depth research on specific upstream and downstream regulatory relationships and patterns. Therefore, in the future, we will propose new ideas for the molecular mechanism of the occurrence and development of obese T2DM with dyslipidemia and provide new research ideas for its diagnosis and treatment through in-depth research.

## Conclusion

(1) The characteristic differences between obese T2DM patients with dyslipidemia and healthy controls are manifested in the significant upregulation of pathways such as fatty acid degradation and significant downregulation of pathways such as glycolysis/gluconeogenesis and pyruvate metabolism. It is speculated that XLOC-005590 and HNF1A-AS1 are potential biomarkers of obese T2DM with dyslipidemia. (2) It is inferred that XLOC-005590 is a potential therapeutic target biomarker for JTTZR intervention in obese T2DM with dyslipidemia. A possible mechanism is that XLOC-005590 reversely regulates the expression of ECI2. ECI2 affects peroxisome and fatty acid degradation pathways. This may be one of the mechanisms underlying JTTZR intervention in obesity, T2DM, and dyslipidemia.

## Data Availability Statement

The datasets presented in this study can be found in online repositories. The names of the repository/repositories and accession number(s) can be found in NCBI GSE193626.

## Ethics Statement

The studies involving human participants were reviewed and approved by This study was approved by the Ethics Committee of Guang’anmen Hospital of China Academy of Chinese Medical Sciences. All participants provided written informed consent. The patients/participants provided their written informed consent to participate in this study.

## Author Contributions

TB, SW, YY, and LHe contributed equally to this study. FL, LZ, and XT conceived and supervised this study. TB, SW, and YY analyzed the data and wrote the original draft. LZ, SW, LHe, TB and LHan designed the study methods. SW, QZ and XZ collected the data. JC and TZ collected the references. All authors have read and approved the final manuscript.

## Funding

This study was supported by the Scientific Specialized Program of Traditional Chinese Medicine of China (No. 201007004) and the Innovation Team and Talents Cultivation Program of National Administration of Traditional Chinese Medicine. (No: ZYYCXTD-D-202001).

## Conflict of Interest

The authors declare that the research was conducted in the absence of any commercial or financial relationships that could be construed as a potential conflict of interest.

## Publisher’s Note

All claims expressed in this article are solely those of the authors and do not necessarily represent those of their affiliated organizations, or those of the publisher, the editors and the reviewers. Any product that may be evaluated in this article, or claim that may be made by its manufacturer, is not guaranteed or endorsed by the publisher.
