# Supplementary material for: Exploring the Regulation of Jiangtang Tiaozhi Formula on the Biological Network of Obese T2DM Complicated With Dyslipidemia Based on Clinical Transcriptomics
Source: Front Endocrinol (Lausanne). 2022 Jul 25;13:817147. doi: 10.3389/fendo.2022.817147 (PMC9357946; doi:10.3389/fendo.2022.817147)
Supplement: Supplementary file 1 [file DataSheet_1.pdf]

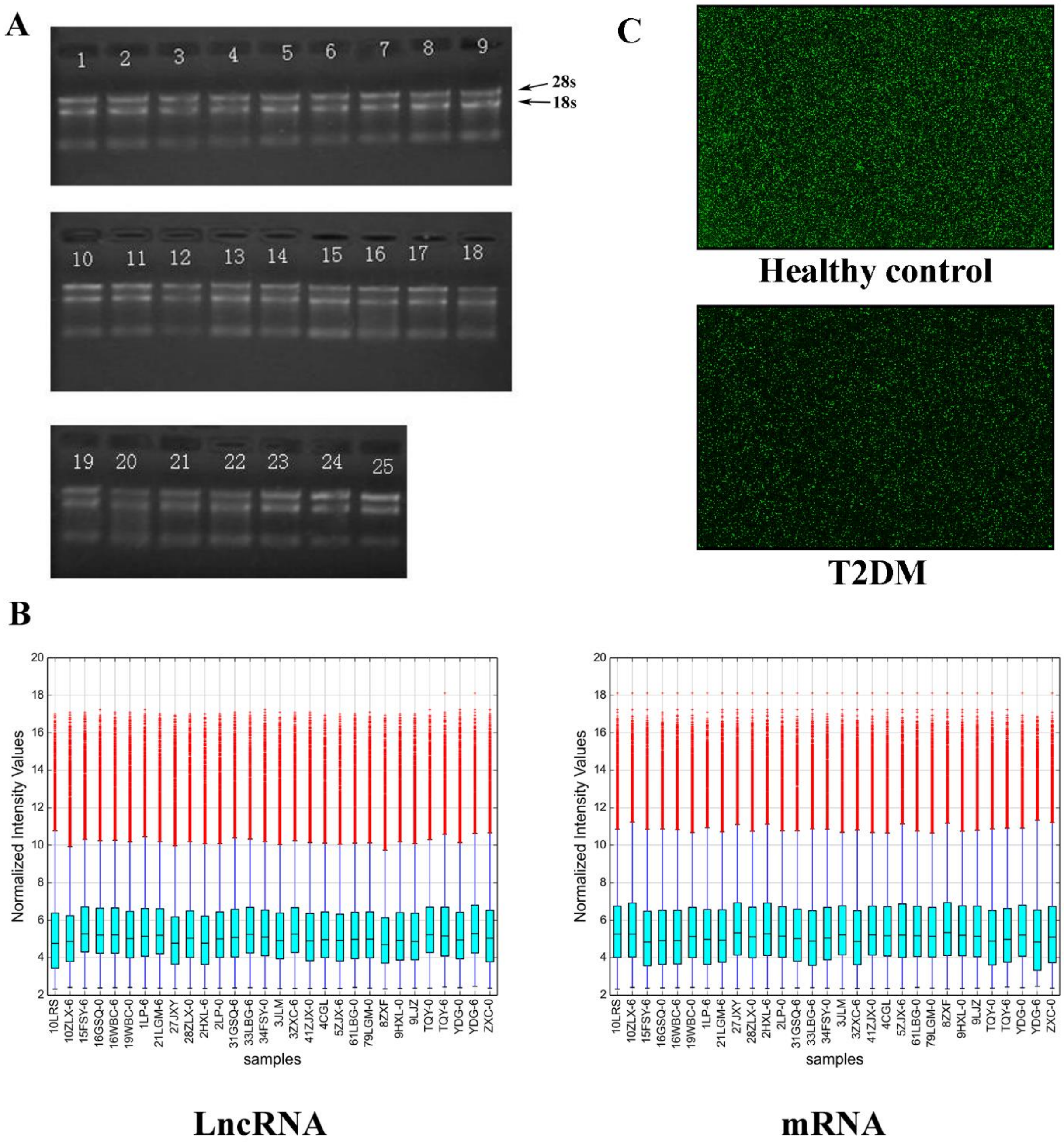

Figure S1 The quality control of RNA and chip results (A: RNA electrophoresis; B: FPKM box diagram. The abscissa is the sample name, and the ordinate is log10 (FPKM+1). The box plot of each area is for five statistics, from top to bottom are the maximum, upper quartile, median, lower quartile and minimum; C: Gene chip scan, taking 1 healthy control group and 1 T2DM group as examples.)
